# Emergence of Hyper-Resistant *Escherichia coli* MG1655 Derivative Strains after Applying Sub-Inhibitory Doses of Individual Constituents of Essential Oils

**DOI:** 10.3389/fmicb.2016.00273

**Published:** 2016-03-04

**Authors:** Beatriz Chueca, Daniel Berdejo, Nelson J. Gomes-Neto, Rafael Pagán, Diego García-Gonzalo

**Affiliations:** ^1^Tecnología de los Alimentos, Departamento de Producción Animal y Ciencia de los Alimentos, Facultad de Veterinaria, Instituto Agroalimentario de Aragón, Universidad de Zaragoza-CITAZaragoza, Spain; ^2^Laboratory of Food Microbiology, Department of Nutrition, Health Sciences Center, Federal University of ParaíbaJoão Pessoa, Brazil

**Keywords:** carvacrol, citral, limonene oxide, mutagenesis, genotypic resistance, filamentation

## Abstract

The improvement of food preservation by using essential oils (EOs) and their individual constituents (ICs) is attracting enormous interest worldwide. Until now, researchers considered that treatments with such antimicrobial compounds did not induce bacterial resistance via a phenotypic (i.e., transient) response. Nevertheless, the emergence of genotypic (i.e., stable) resistance after treatment with these compounds had not been previously tested. Our results confirm that growth of *Escherichia coli* MG1655 in presence of sub-inhibitory concentrations of the ICs carvacrol, citral, and (+)-limonene oxide do not increase resistance to further treatments with either the same IC (direct resistance) or with other preservation treatments (cross-resistance) such as heat or pulsed electric fields (PEF). Bacterial mutation frequency was likewise lower when those IC's were applied; however, after 10 days of re-culturing cells in presence of sub-inhibitory concentrations of the ICs, we were able to isolate several derivative strains (i.e., mutants) displaying an increased minimum inhibitory concentration to those ICs. Furthermore, when compared to the wild type (WT) strain, they also displayed direct resistance and cross-resistance. Derivative strains selected with carvacrol and citral also displayed morphological changes involving filamentation along with cell counts at late-stationary growth phase that were lower than the WT strain. In addition, co-cultures of each derivative strain with the WT strain resulted in a predominance of the original strain in absence of ICs, indicating that mutants would not out-compete WT cells under optimal growth conditions. Nevertheless, growth in the presence of ICs facilitated the selection of these resistant mutants. Thus, as a result, subsequent food preservation treatments of these bacterial cultures might be less effective than expected for WT cultures. In conclusion, this study recommends that treatment with ICs at sub-inhibitory concentrations should be generally avoided, since it could favor the emergence of hyper-resistant strains. To ascertain the true value of EOs and their ICs in the field of food preservation, further research thus needs to be conducted on the induction of increased transient and stable bacterial resistance via such antimicrobial compounds, as revealed in this study.

## Introduction

Food-related bacteria have developed strategies to overcome a wide variety of unfavorable environmental conditions encountered during food processing or storage: extremely low or high temperatures (Vidovic et al., 2011, 2012), acid pH and a high amount of salts (McMahon et al., 2007), pulsed electric fields (PEF; Sagarzazu et al., 2013), high hydrostatic pressure (HHP; Hauben et al., 1997), and chemical preservatives (Patrignani et al., 2008; Dubois-Brissonnet et al., 2011). These strategies can lead to an increase in bacterial resistance to the homologous stress agent (direct resistance) as well as to heterologous environmental stresses (cross-resistance; Hengge-Aronis, 2011). An increase in bacterial resistance can follow two different patterns: phenotypic resistance, which is transient and under metabolic control, and genotypic resistance, which results from genetic mutations and thus has a permanent effect (Charpentier and Tuomanen, 2000; Corona and Martínez, 2013).

The development of resistance to antibiotics is usually associated with an inheritable resistance that requires a genetic modification, or its acquisition by horizontal gene transfer (Corona and Martínez, 2013). Kohanski et al. (2010) demonstrated a correlation between the mutagenesis induced by sublethal concentrations of some bactericidal antibiotics and an increase in reactive oxygen species (ROS). This pathway is dependent on *recA*, which induces SOS response-mutagenesis (Thi et al., 2011).

Recent studies have suggested that essential oils (EOs) or their individual constituents (ICs) have little impact on the development of antimicrobial resistance (McMahon et al., 2008; Hammer et al., 2012; Luz et al., 2012a,b; Gomes Neto et al., 2015). Although plants and their EOs have been long recognized as a valuable source of medicinal agents (Bakkali et al., 2008), only recently have they been proposed as alternatives to help control spoiling and pathogenic microorganisms in the food industry (Burt, 2004; Friedman, 2015). Carvacrol, citral, and (+)-limonene are monoterpenes generally recognized as safe (GRAS) food additives whose antimicrobial activities and mechanisms of inactivation have been examined (Somolinos et al., 2010; Ait-Ouazzou et al., 2013; Chueca et al., 2014a,b; Espina et al., 2014). As described in the case of bactericidal antibiotics by Kohanski et al. (2007), an ROS-dependent mechanism of *Escherichia coli* inactivation was demonstrated for lethal treatments of carvacrol, citral and (+)-limonene (Chueca et al., 2014a,b). Moreover, RecA-mediated SOS response is a commonly activated pathway in bactericidal antibiotics, carvacrol, citral and (+)-limonene: thus, after sublethal treatments with those ICs, one could expect that a mutagenesis would lead to emergence of hyper-resistant mutants.

Although the prospect that food-related bacteria would develop phenotypic resistance after exposure to sub-inhibitory concentrations of EOs and ICs has been ruled out (Luz et al., 2012a,b; Gomes Neto et al., 2015), studies about the possible induction of genotypic bacterial resistance are still scarce.

IC-induced phenotypic and genotypic resistance to food preservation treatments, both chemical (to ICs or food preservatives) and physical (to heat, PEF or HHP), should be evaluated to ascertain the value of ICs as new antimicrobial agents in this field. The resulting findings could likewise help us design combined food preservation processes more efficiently.

This study's objectives are (a) to investigate the effect of *Escherichia coli* MG1655 growth in presence of sub-inhibitory concentrations of carvacrol, citral, and (+)-limonene oxide on the development of phenotypic and genotypic resistance to chemical (ICs) and physical treatments (heat and PEF); and (b) to determine the cell morphology and growth properties of resulting hyper-resistant derivative strains.

## Materials and methods

### Micro-organisms and growth conditions

The strain used in this study was *Escherichia coli* MG1655. The culture was maintained in a cryovial at −80°C, from which plates of tryptic soy agar (Oxoid, Basingstoke, Hampshire, England) with 0.6% yeast extract (Oxoid; TSAYE) were prepared on a weekly basis.

We prepared broth subcultures by inoculating a test tube containing 5 mL of sterile tryptic soy broth (Oxoid) and 0.6% yeast extract (TSBYE) with one single colony from a plate. After inoculation, the tubes were incubated overnight at 37°C. 250 mL-flasks containing 50 mL of TSBYE were inoculated with the resulting subcultures to a final concentration of 10^5^ colony-forming units (CFU)/mL. These flasks were incubated under agitation (130 rpm; Selecta, mod. Rotabit, Barcelona, Spain) at 37°C until late-stationary growth phase was reached (24 h).

### Minimum inhibitory concentration (MIC)

MIC against *E. coli* MG1655 was determined for carvacrol (95%; Sigma-Aldrich, Steinheim, Germany), citral (95%; Sigma-Aldrich), and (+)-limonene oxide (97%; Sigma-Aldrich) by the tube dilution method with an initial concentration of 10^5^ CFU/mL (Rota et al., 2004). The highest and lowest concentrations tested were 1750 and 50 μl/L of ICs, respectively. These ICs are practically immiscible in water: therefore, we applied a vigorous shaking method to prepare suspensions (Friedman et al., 2002). For ICs, we also prepared negative controls containing TSBYE plus 1750 μl/L of ICs, and positive controls containing TSBYE with microorganisms at a final concentration of 10^5^ CFU/mL. After 24 h incubation at 37°C, the MIC was determined as the lowest concentration of each IC in the presence of which bacteria failed to grow, i.e., at which no visible changes could be detected in the broth medium (Clinical and Laboratory Standards Institute, 2012).

### Pre-exposure of wild type (WT) *E. coli* strain to ICs to evaluate the emergence of phenotypic resistance

The assays to evaluate phenotypic changes in bacterial resistance after exposure of *E. coli* MG1655 to sub-inhibitory (sub-MIC) concentrations of carvacrol, citral, or (+)-limonene oxide in TSBYE were performed following a procedure described by Gomes Neto et al. (2012). Briefly, 2 mL of broth subculture were inoculated in 18 mL of TSBYE containing sub-MIC concentrations (between ½ and ¾ × MIC) of carvacrol, citral, or (+)-limonene oxide in tubes and vortexed for 30 s (initial cell counts at ca. 10^7^ CFU/mL). The tubes were incubated under agitation (130 rpm) for 24 h at 37°C. After the incubation period, lethal treatments with ICs, heat and PEF were performed, as explained below.

### Pre-exposure of wild type (WT) *E. coli* strain to ICs to evaluate the emergence of genotypic resistance

We selected derivative strains of *E. coli* MG1655 following the procedure described in Kohanski et al. (2010) for bactericidal antibiotics. An overnight culture of *E. coli* was diluted 1:10,000 into 50 mL TSBYE in a 250 mL-flask and grown for 3.5 h at 37°C and 130 rpm. This second culture was diluted 1:3 into fresh TSBYE containing sub-MIC concentrations (between ½ and ¾ × MIC) of carvacrol, citral, or (+)-limonene oxide. Five milliliter of these diluted cultures were grown in tubes for 24 h at 37°C and 130 rpm. Each day thereafter for 10 days, cells were diluted 1:1000 into 5 mL TSBYE in a tube containing a sub-MIC amount of the respective IC and grown for 24 h at 37°C and 130 rpm. After 10 days, 0.1 mL-samples of each culture were pour-plated and six colonies were selected for re-growth in fresh TSBYE to verify that the changes in resistance observed were due to stable genotypic alterations in the new strains and to determine their MIC for the selection agent ICs.

The exposure of bacterial cells to an agent that increases mutation beyond the normal rate also raises the chances that cell function might be altered (Giraud et al., 2001). We investigated each IC's mutagenic effect by evaluating the occurrence of mutants resistant to rifampicin as a result of point mutations in the *rpoB* gene (Gutierrez et al., 2013). To achieve this, we started by diluting an overnight culture of *E. coli* 1:10,000 into 50 mL TSBYE in a 250 mL-flask; it was grown for 3.5 h at 37°C under agitation (130 rpm). The culture was then diluted 1:3 into fresh TSBYE containing sub-MIC concentrations of carvacrol, citral, or (+)-limonene oxide (at a final concentration of ¾ × MIC) or 1 mg/L ampicillin (Sigma-Aldrich) as a positive control. 20 mL-aliquots (five replicates) of these diluted cultures were grown in 100 mL-flasks for 24 h at 37°C and 130 rpm, with absorbance at 595 nm similar for all cultures, in order to obtain detectable and comparable mutant frequencies (Kohanski et al., 2010). Aliquots of each flask were serially diluted and plated on TSAYE with and without 100 mg/L rifampicin (Sigma-Aldrich). Plates were incubated for 48 h at 37°C, and CFU were counted. Mutation rates were determined by dividing the number of CFU grown in rifampicin plates (mutation events) by the number of CFU grown in antibiotic-free plates (Rosche and Foster, 2000). Fold change in mutation rate was determined for each IC relative to an untreated control.

### Evaluation of increased bacterial resistance

#### Lethal IC treatments

Prior to treatment, cultures were centrifuged at 6000 × *g* for 5 min and resuspended at a final concentration of 2 × 10^7^ CFU/mL in 10 mL of McIlvaine citrate-phosphate buffer of pH 7.0 with 200 μL/L of carvacrol, 300 μL/L of citral, or 500 μL/L of (+)-limonene oxide added. These treatment conditions were chosen according to preliminary results (data not shown). IC treatments were carried out at room temperature. 0.1 mL samples were taken at determined intervals (15, 20, or 60 min for carvacrol, citral, or (+)-limonene oxide, respectively) to enumerate survivors.

#### Lethal heat treatments

Heat treatment was carried out in an incubator (FX Incubator, mod. ZE/FX, Zeulab, Zaragoza, Spain) at 55°C, with a thermocouple (Ahlborn, mod. Almemo 2450, Holzkirchen, Germany) to monitor heating temperature. Prior to treatment, cultures were centrifuged at 6000 × g for 5 min and resuspended at a final concentration of 2 × 10^7^ CFU/mL in 450 μL of McIlvaine citrate-phosphate buffer of pH 7.0 once temperature had stabilized at 55°C. These treatment conditions were chosen according to preliminary results (data not shown). After treating bacterial suspensions at that temperature for 26 min, 0.1 mL-samples were taken to enumerate the amount of survivors.

#### Lethal PEF treatments

PEF treatments were carried out using equipment that delivers exponential decay pulses, as described by García et al. (2005). High electric field pulses were produced by discharging a set of 10 capacitors (6800 pF; Behlke, C-20C682, Kronberg, Germany) via a thyristor switch (Behlke HTS 160-500SCR) in a batch treatment chamber. The capacitors were charged by a high-voltage DC power supply (FUG, HCK 2500M 35,000, Rosenhein, Germany); a function generator (Tektronix AFG 320, Wilsonville, OR, USA) delivered the on-time signal to the switch. The treatment chamber consisted of a cylindrical plastic tube closed with two polished stainless steel electrodes (Raso et al., 2000). The gap between electrodes was 0.25 cm, and the electrode area was 2.01 cm^2^. Voltage and electrical intensity were measured with a high voltage probe and a current probe, each connected to an oscilloscope (Tektronix TDS 3012B). Experiments began at room temperature (22 ± 2°C), and temperature never exceeded 35°C inside the treatment chamber. Prior to this treatment, microorganisms were centrifuged at 6000 × *g* for 5 min and resuspended for a final concentration of approximately 2 × 10^7^ CFU/mL in McIlvaine citrate-phosphate buffer of pH 7.0 while electrical conductivity was adjusted to 2 mS/cm. Next, 0.5 mL of the samples were introduced in the treatment chamber with a sterile syringe, as described by Raso et al. (2000). Cell suspensions were treated with 50 pulses (1 Hz, pulse width 2 μs) at 35 kV/cm, corresponding to a specific charge of 4.06 kJ/kg per pulse. These treatment conditions were chosen according to preliminary results (data not shown). After treatments, 0.1 mL-samples were taken to enumerate survivors.

#### Enumeration of survivors

After treatment, samples were diluted in phosphate buffered saline, pH 7.3 (Oxoid). Then 0.1 mL-samples were pour-plated onto TSAYE. Plates were incubated at 37°C for 24 h. After plate incubation, the colonies were counted with an improved image analysis automatic colony counter (Protos; Analytical Measuring Systems, Cambridge, United Kingdom) as described by Condón et al. (1996). Inactivation was expressed as the difference in log_10_ counts prior and posterior to treatment in each case. The error bars in the figures indicate the mean ± standard deviations from the data obtained from at least three independent experiments carried out with different microbial cultures.

### Determination of cell morphology of WT and derivative strains

We observed derivative and WT strain cells grown in absence of ICs with a phase contrast microscope (Nikon Eclipse E400, Nikon Corporation, Tokyo, Japan). Two independent cultures were grown at 37°C for 24 h for each strain. We used a high-resolution AxioCam MRc camera (Carl Zeiss AG, Oberkochen, Germany) to capture digital images that were subsequently processed by ZEN 2010 software (Carl Zeiss AG).

### Growth properties of WT and derivative strains

Growth was monitored with an absorbance microplate reader (Tecan Ltd., Tecan Genios, Seestrasse, Switzerland) to measure bacterial culture absorbance at 595 nm during incubation. To characterize growth kinetics, absorbance values were fitted using Gompertz model nonlinear regression (Gibson et al., 1988), which in this case can be described as follows:

(1) A(t)=C×exp(−exp(−B×(t−M)))

where *A(t)* is the absorbance value in time *t, C* is the absorbance value in the stationary phase, *B* is the relative growth rate in point *M*, and *M* is the time at which the cells reach their maximum growth rate.

### Competition experiments between WT and derivative strains

In a competition experiment to evaluate growth capacity of the mutants as compared with that of the WT (i.e., fitness), each derivative strain was grown along with the WT in the same tube. To differentiate each strain within the co-culture, one of them was marked with a kanamycin resistance cassette. Knockouts of *malE* were constructed using P1 phage transduction and were derived from an *E. coli* single-gene knockout library (Baba et al., 2006). Positive P1 transductants were confirmed by acquisition of kanamycin resistance. We generated cells of WT and derivative strains containing the kanamycin cassette so that we could account for fitness effects of the cassette itself. Strain behavior in the competition assays after *malE* deletion or kanamycin resistance cassette insertion did not present any observable modification (data not shown).

Co-cultures of WT and each derivative strain were produced by diluting overnight cultures 1:10,000 into 50 mL of TSBYE, or TSBYE added with sub-MIC concentrations (between ½ and ¾ × MIC) of carvacrol, citral or (+)-limonene oxide, in a 250 mL-flask and grown at 37°C and 130 rpm. To estimate differences in population fitness via the evaluation of respective population sizes, we plated appropriately diluted samples of the co-culture onto non-selective (TSAYE) and selective growth media (TSAYE with 30 mg/L of Sigma-Aldrich kanamycin added), thereby allowing the growth of one competitor—the marked strain, resistant to kanamycin—while precluding the growth of the other.

For each competition experiment we computed the derivative strain's relative fitness (*W*_*der*_) by comparing the *n*-fold expansion of the derivative and WT strains (Lenski et al., 1991) as follows:

(2) $$W_{der\;}=\left\{\text{ln}\left[N_{der}\left(T\right)\times d/N_{der}\left(0\right)\right]/\text{ln}\left[N_{w}(\text{T})\times d/N_{w}\left(0\right)\right]\right\}$$

in which *N*(0) and *N*(T) are the proportions of the WT (*N*_*w*_) and derivative strain (*N*_*der*_) in the population at the onset (0) and at the end (T) of the competition respectively, and *d* represents entire population growth during the competition:

(3) $$\begin{array}{l} d=\{[\left(\text{CFU}/\text{mL}\right)\;\text{ of all bacteria at T}]/[\left(\text{CFU}/\text{mL}\right)\text{of all}\\ \text{ bacteria at }0]\} \end{array}$$

The relative fitness (*W*_*der*_) of a derivative strain is a dimensionless factor that measures the growth rate of the derivative strain compared to the WT within a specific cellular environment.

### Statistical analysis

Data for the evaluation of the MIC, mutation rate experiments, lethal treatments and competition experiments were obtained from at least three independent experiments carried out with different microbial cultures.

ANOVA and *t*-tests were performed with GraphPad PRISM® (GraphPad Software, Inc., San Diego, USA) and differences were considered significant if *p* ≤ 0.05.

## Results

### ICs do not increase phenotypic resistance

In order to evaluate the emergence of phenotypic resistance after pre-exposure of WT *E. coli* MG1655 to ICs, cells were incubated with sub-MIC concentrations of carvacrol, citral, or (+)-limonene oxide added to the growth medium until bacterial cultures reached late-stationary growth phase (24 h). These cells were then treated with the same IC applied during their growth (direct resistance), and no differences were found between the resistance of pre-exposed and non-pre-exposed cells (*p* > 0.05; data not shown). Pre-exposed cells were also subjected to lethal treatments with the remaining ICs, as well as with physical food preservation treatments—specifically, heat and PEF (cross-resistance). Just as observed in the evaluation of direct resistance, cells did not display increased phenotypic cross-resistance after having been pre-exposed to an IC (*p* > 0.05; data not shown).

### Isolation of *E. coli* strains with increased genotypic resistance to ICs

After a 10-day selection with sub-MIC concentrations of carvacrol, citral, and (+)-limonene oxide, we isolated *E. coli* derivative strains. Six strains from each type of selection were randomly selected and re-cultured in TSBYE. In order to evaluate each derivative strain's increase in resistance, an MIC assay was performed. As shown in Table 1, MICs for WT (i.e., the original strain), were 200 μL/L of carvacrol, 1000 μL/L of citral, and 750 μL/L of (+)-limonene oxide. On the one hand, after selection with either citral or (+)-limonene oxide, the six evaluated colonies showed the same increase in resistance, with MICs of >1750 and 1500 μL/L for citral and (+)-limonene oxide, respectively. On the other hand, in the presence of carvacrol as selection agent, only three of the six colonies became more resistant, displaying an MIC of 600 μL/L for that specific IC. From the growth at sub-MIC concentrations of carvacrol, citral, and (+)-limonene oxide, we respectively selected three derivative strains showing increased genotypic resistance: CAR_1_ CIT_1_ and LIM_1_ (which from now on we will call CAR, CIT, and LIM).

**Table 1:** **Minimum inhibitory concentration (MIC; μL/L) of *Escherichia coli* MG1655 wild type strain (WT) and its derivative strains obtained by selection with carvacrol (CAR_1_–CAR_6_), citral (CIT_1_–CIT_6_), and (+)-limonene oxide (LIM_1_ to LIM_6_)**.

**Strains tested**	**Carvacrol**	**Citral**	**(+)-Limonene oxide**
WT	200	1000	750
CAR_1_–CAR_3_	600	n.d.	n.d.
CAR_4_–CAR_6_	200	n.d.	n.d.
CIT_1_–CIT_6_	n.d.	>1750	n.d.
LIM_1_–LIM_6_	n.d.	n.d.	1500

*n.d., not determined*.

**Figure 1:**
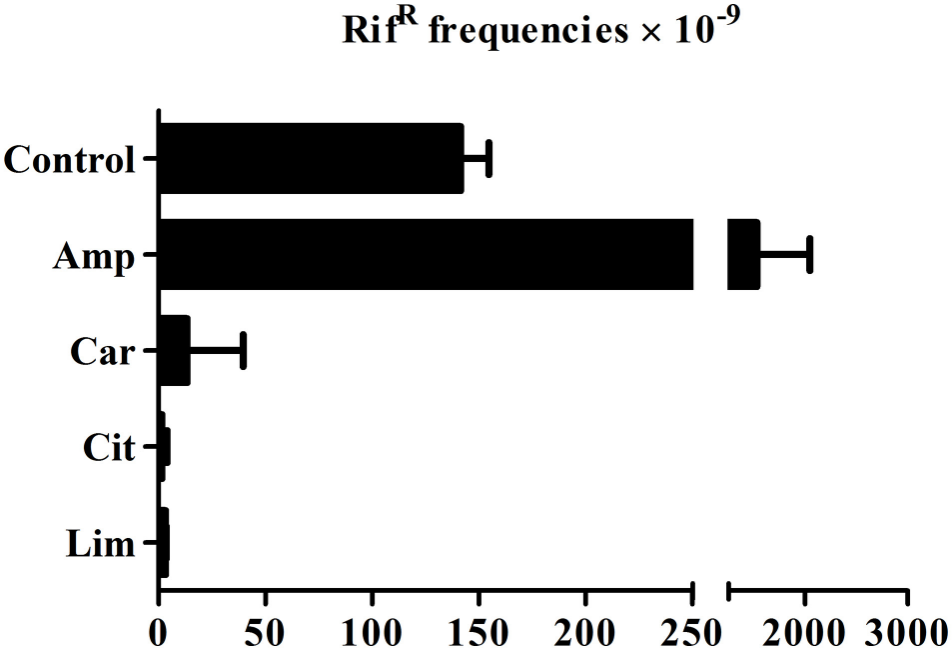
**Induced mutagenesis in *Escherichia coli* MG1655 in presence or absence of carvacrol, citral, (+)-limonene oxide, and ampicillin**. Bacteria were grown in tryptic soy broth with yeast extract added, supplemented with 150 μL/L carvacrol (Car), 750 μL/L citral (Cit), 562.5 μL/L (+)-limonene oxide (Lim) and 1 mg/L ampicillin (Amp) or no compound added (control). The frequency of the rifampicin-resistant (Rif^R^) mutants was measured. Data are means ± standard deviations (error bars).

In order to evaluate the role of ICs in the emergence of genotypic resistance, we determined mutation rates using a rifampicin-based selection method, focusing on the accumulation of mutations that confer resistance to rifampicin due to a mutagenic agent. We thus compared mutation rates observed in *E. coli* MG1655 after 24 h of growth in TSBYE in presence and absence of ICs. Firstly, this strain displayed a spontaneous frequency of rifampicin-resistant mutants of nearly 2 × 10^−7^, meaning that 2 out of 10^7^ cells had developed resistance to rifampicin. Mutant frequencies after incubation with carvacrol, citral and (+)-limonene oxide were at least 10 times lower than the frequency observed in absence of any IC (Figure 1). However, presence of ampicillin entailed a 10-fold increase in mutant frequency.

### Evaluation of direct- and cross-resistance of selected derivative strains to lethal treatments with carvacrol, citral, (+)-limonene oxide, heat, and PEF

**Figure 2:**
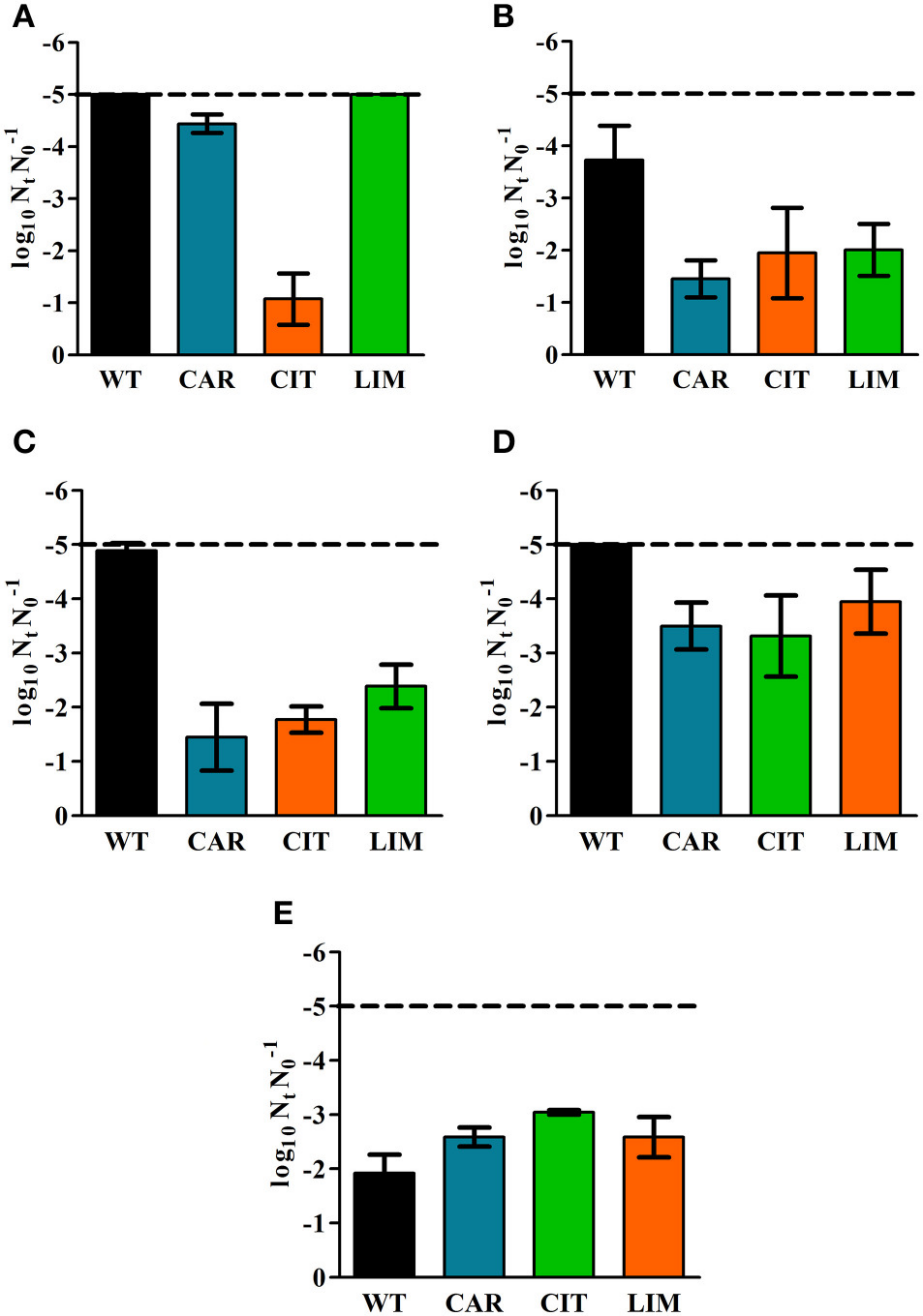
**Inactivation of *Escherichia coli* MG1655 wild type strain (WT) and its derivative strains, obtained by selection with carvacrol (CAR), citral (CIT) and (+)-limonene oxide (LIM), after lethal treatments with: 200 μL/L of carvacrol during 15 min (A); 300 μL/L of citral during 20 min (B); 500 μL/L of (+)-limonene oxide during 60 min (C); heat treatment at 55°C during 26 min (D); and a PEF treatment at 35 kV/cm for 50 pulses (E)**. Treatment medium was citrate-phosphate buffer of pH 7.0. Data are means ± standard deviations (error bars).

Once derivative strains had been selected, we evaluated their resistance to several chemical compounds (ICs) and to other physical food preservation technologies (heat and PEF) in citrate-phosphate buffer of pH 7.0 with an initial concentration of 2 × 10^7^ CFU/mL. Figure 2A shows the inactivation of WT and derivative strains provoked by a treatment with 200 μL/L of carvacrol. A similar inactivation of ca. 5 log_10_ cycles was observed for WT, CAR and LIM (*p* > 0.05). However, the CIT strain was more resistant than the others (*p* ≤ 0.05).

Regarding resistance against 300 μL/L of citral, the three derivative strains showed a higher resistance than the WT strain (*p* ≤ 0.05; Figure 2B). While nearly 4 log_10_ cycles of the WT strain had been inactivated after 20 min, only ca. 2 log_10_ cycles of CAR, CIT, and LIM cells were killed under the same conditions.

All of the derivative strains obtained in this study were more resistant to a 60 min-treatment with 500 μL/L of (+)-limonene oxide than the WT strain (*p* ≤ 0.05; Figure 2C). This treatment inactivated 5 log_10_ cycles of WT cells, but only 2 log_10_ cycles of the initial population of CAR, CIT, and LIM cells.

As can be seen in Figure 2D, the CAR, CIT, and LIM strains were more heat-resistant than the WT strain after a 26-min-treatment at 55°C (*p* ≤ 0.05). Inactivation due to heat surpassed the detection limit (5 log_10_ cycles) in the WT strain, whereas CAR, CIT, and LIM displayed around 4 log_10_ cycles of inactivation.

Evaluation of PEF resistance showed that ca. 2 log_10_ cycles of initial bacterial populations of WT, CAR, and LIM strains were inactivated after 50 pulses at 35 kV/cm (*p* > 0.05). Inactivation of the derivative strain CIT by PEF was 1 log_10_ cycle higher than that of the WT strain (*p* ≤ 0.05; Figure 2E).

### Determination of cell morphology and growth properties of the hyper-resistant derivative strains

**Figure 3:**
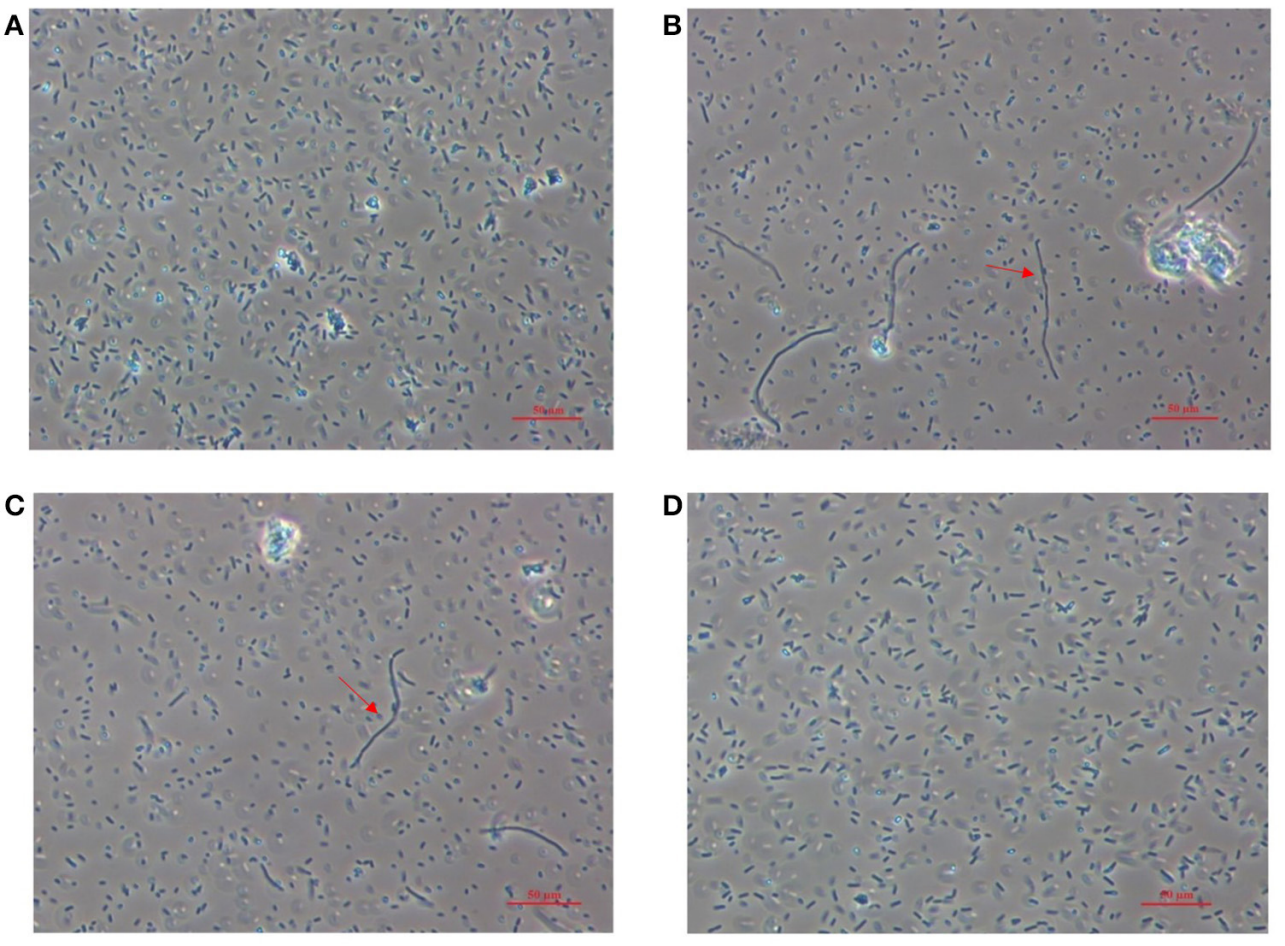
**Microscopic image obtained from bacterial cultures of *Escherichia coli* MG1655 wild type strain (A) and its derivative strains, obtained by selection with carvacrol (B); citral (C); and (+)-limonene oxide (D)**. Cultures were obtained after 24 h of incubation at 37°C in tryptic soy broth with yeast extract added. Arrows indicate examples of the morphology of filamentous cells.

Microscopic comparison of the hyper-resistant derivative strains with the WT strain revealed differing morphologies in the CAR and CIT cell populations, but not in the LIM strain. As seen in Figure 3, around 5% of CAR and CIT cells were longer, yet similar in width to the WT cells, a phenomenon commonly known as filamentation.

**Figure 4:**
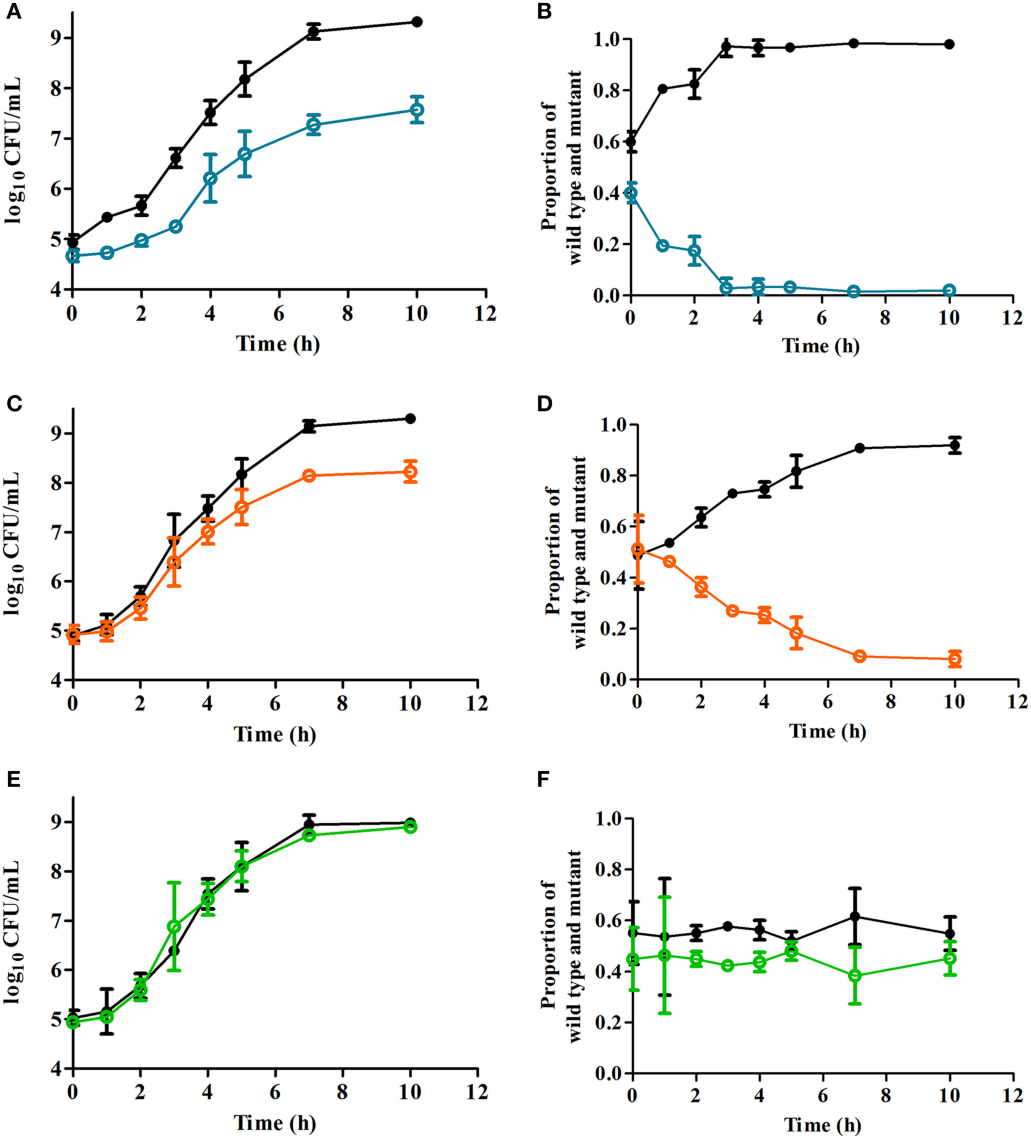
**Growth curves measured as log_10_ CFU/mL (A,C,E) and proportion of strains within the entire population (B,D,F) of co-cultures with *Escherichia coli* MG1655 wild type strain (∙) and its derivative strains obtained by selection with carvacrol (○, A,B); citral (○, C,D); or (+)-limonene oxide (○, E,F)**. Cultures were obtained at 37°C in tryptic soy broth with yeast extract added. Data are means ± standard deviations (error bars).

As shown in Table 2, although growth rates for all strains were similar (*p* > 0.05), CFU counts in late-stationary growth phase were lower for single cultures of CAR and CIT strains (≤ 1 × 10^9^ CFU/mL), amounting to at least half of the cell counts obtained for WT and LIM strains (2 × 10^9^ CFU/mL). In order to learn more about the growth properties of derivative strains, we evaluated co-cultures of WT with each derivative strain starting at 10^5^ CFU/mL for each strain in liquid growth medium in absence of ICs (Figures 4A,C,E). Statistical differences (*p* ≤ 0.05) in cell counts between WT strain and both CAR and CIT emerged starting with 1 and 7 h of incubation respectively (Figures 4A,C).

**Table 2:** **Growth rates and late-stationary phase CFU/mL counts for *Escherichia coli* MG1655 wild type strain (WT) and its derivative strains obtained by selection with carvacrol (CAR), citral (CIT), and (+)-limonene oxide (LIM) grown at 37°C for 24 h in tryptic soy broth with yeast extract added**.

**Strain**	**Growth rate**	**Late-stationary growth phase (24 h) CFU/mL**
WT	1.06 ± 0.14^1^	2.45 × 10^9^ ± 2.95 × 10^8^^a^
CAR	0.86 ± 0.12^1^	1.61 × 10^9^ ± 2.14 × 10^8^^b^
CIT	1.45 ± 0.50^1^	8.41 × 10^8^ ± 9.06 × 10^7^^c^
LIM	1.31 ± 0.30^1^	2.26 × 10^9^ ± 3.19 × 10^8^^a^

*Data are means of three independent observations ± standard deviations*.

a,b,c same letters or

1 *numbers indicate non-significant differences among mean values; p > 0.05. Growth rates and CFU/mL were studied independently*.

Figures 4B,D,F represent the proportion of WT and derivative strains in each co-culture. Whereas LIM and WT proportions after 10 h of co-culture were similar (Figure 4F), CAR and CIT cells represented only 0.02 and 0.08% of the final population respectively (Figures 4B,D). The relative fitness *W*_*der*_ of a mutant is a dimensionless factor that measures the mutant's growth rate compared to WT within a specific cellular environment. Table 3 shows that, whereas *W*_*der*_ for LIM is nearly 1 (indicating that LIM and WT display similar relative fitnesses), CAR and CIT strains are less efficient than WT when cultured together.

**Table 3:** **Relative fitness (*W_der_*) of *Escherichia coli* MG1655 derivative strains obtained by selection with carvacrol (CAR), citral (CIT) and (+)-limonene oxide (LIM)**.

**Co-culture**	***W_der_***	***W_der_* (ICs)**
WT + CAR	0.66^a^ ± 0.04	0.74^a^ ± 0.15
WT + CIT	0.75^a^ ±0.01	1.36^b^ ± 0.05
WT + LIM	0.95^a^ ± 0.05	1.10^b^ ± 0.06

Cells from derivative strains were grown in co-culture with wild type cells (WT) at 37°C for 10 h in tryptic soy broth with yeast extract added, in absence (*W_der_*) and presence of the same individual constituent (ICs) used as selection agents [*W_der_*(ICs)]. Data are means of three independent observations ± standard deviations. Co-cultures involving the same strains were studied independently, thus

a,b *same letters in each line indicate non-significant differences among mean values; p > 0.05*.

However, when carvacrol at sub-MIC concentrations was added to the growth medium, relative fitness of CAR was not significantly affected (*p* > 0.05). In contrast to results observed for CAR, co-culture of CIT and WT under the presence of citral revealed that the CIT strain's fitness was higher than that of WT cells (*p* ≤ 0.05), with CIT becoming the major strain in that co-culture and displaying a *W*_*der*_ of 1.36 (Table 3). Although *W*_*der*_ of the LIM strain was likewise higher in presence of (+)-limonene oxide, its increase was lower than *W*_*der*_ of the CIT strain. Consequently, LIM would also out-compete the WT strain in presence of the IC used during the selection of the hyper-resistant derivative strain.

## Discussion

This study has demonstrated, for the first time, the emergence of hyper-resistant strains after exposure to the ICs carvacrol, citral, and (+)-limonene oxide. Their resistance was stable after re-growth in absence of the antimicrobial compounds, thus it can be attributed to genotypic modifications of the original strain. In addition, to the best of our knowledge, this is the first report of bacterial resistance against lethal treatments with (+)-limonene oxide.

At the onset, increased phenotypic resistance was evaluated after 24 h bacterial growth in the presence of sublethal concentrations of carvacrol, citral and (+)-limonene oxide. No changes in resistance to either chemical (ICs) or physical (heat and PEF) lethal treatments were observed, in agreement with previous studies (McMahon et al., 2008; Hammer et al., 2012; Luz et al., 2012a,b; Gomes Neto et al., 2015). As a consequence, no increased phenotypic direct- or cross-resistance was detected after pre-exposing *E. coli* cells to sub-MIC concentrations of ICs for 24 h.

Therefore we proceeded to evaluate the emergence of genotypic or stable resistance after a 10-day selective exposure to sub-MIC concentrations of ICs. Sub-MIC concentrations of bactericidal antibiotics increased intracellular ROS production (Kohanski et al., 2007), leading to activation of SOS response which caused mutagenesis dependent on RecA activity (Kohanski et al., 2010; Thi et al., 2011). Since ROS were involved in the mechanisms of bacterial inactivation via lethal treatments with carvacrol, citral, and (+)-limonene (Chueca et al., 2014a,b), a mutagenic activity similar to the one usually triggered by bactericidal antibiotics was expected. It should be noted that spontaneous mutations occur at random during bacterial growth under optimal conditions (e.g., in absence of ICs). Evaluation of frequency of mutation for our WT strain in absence of ICs (Figure 1) showed similar values than for ME12, an MG1655 derivative (Thi et al., 2011). We also confirmed that ampicillin increased mutation frequency of WT as described by Kohanski et al. (2010). Contrary to what was expected, none of the tested ICs increased bacterial mutagenesis when compared with optimal growth conditions. A lower mutation frequency in presence of ICs would suggest a protective role of those ICs at sub-MIC concentration—contrary to the expected effect of ICs, which, at lethal concentrations, would activate an SOS response (Chueca et al., 2014a,b). Indeed, although the mechanism of bacterial inactivation through carvacrol, citral and (+)-limonene is related to ROS production at lethal concentrations (Chueca et al., 2014a,b) these compounds are also regarded as natural antioxidants (Brewer, 2011). Likewise, Llana-Ruiz-Cabello et al. (2015) demonstrated that high concentrations of carvacrol induce oxidative stress in the Caco-2 cell line, but low concentrations of the same IC reverse oxidative damage. Consequently, the observed protection from mutation which is conferred by those ICs could be related to their antioxidant activity at the low sub-MIC concentrations which were applied.

However, after 10 days of continuous exposure to sub-MIC concentrations of each IC, we isolated several *E. coli* MG1655 derivative strains which displayed an increased MIC to the selective agent (Table 1); we proceeded to characterize three of those strains (CAR, CIT, and LIM) in further detail. According to Fridman et al. (2014), bacterial strategies against chemical agents such as antibiotics could be divided into those which allow a microorganism to grow in the constant presence of an antibiotic at low concentrations (e.g., higher MICs for inhibition assays), and those strategies permitting a microorganism to survive at high antibiotic concentrations but during a limited time (e.g., a higher survival rate from inactivation treatments). Thus, in order to improve our description of the effects of mutations, we not only verified the increase in MICs of derivative strains and evaluated their resistance to lethal stresses of the same IC applied during selection (direct resistance), but we also measured their resistance to the other ICs as well as to physical treatments (cross-resistance).

We started by evaluating increased direct resistance. On the one hand, when compared with the WT strain, the CIT and LIM strains displayed an increased resistance to lethal treatments with citral and (+)-limonene oxide respectively. On the other hand, although the CAR strain had an increased MIC to carvacrol, it was not more resistant to a lethal treatment with carvacrol than the WT strain (Figure 2A). This situation could indicate that the mechanism of action of carvacrol microbial in inhibition assays might differ from its performance in inactivation treatments.

We then proceeded to assess the development of cross-resistance in derivative strains. First of all, the three derivatives' heat resistance was likewise increased. All three derivative strains displayed increased resistance against citral and (+)-limonene oxide, but only one of them (CIT) developed a higher resistance to carvacrol. However, CAR and LIM derivative strains showed a PEF resistance similar to that observed in the WT strain (*p* > 0.05), and CIT was even less PEF-resistant than the WT (*p* ≤ 0.05). Hauben et al. (1997) correlated the increased heat resistance of barotolerant *E. coli* mutants with similarities between the cellular targets of lethal pressure and heat treatments. Likewise, increase of heat resistance in IC-resistant strains could be related to bacterial envelopes, which are regarded as these preservation technologies' primary targets: heat permeabilizes the outer and cytoplasmic membranes (Tsuchido et al., 1985; Mackey et al., 1991); citral can cause sublethal injuries in the outer and inner membranes of *E. coli* (Somolinos et al., 2010); (+)-limonene can damage the lipopolysaccharide fraction of the cell wall along with the proteins and phospholipids of the outer and inner membranes (Espina et al., 2013); carvacrol, finally, has the capability of interacting with both the lipid bilayer and proteins of the inner and outer membranes (Burt et al., 2007; Hyldgaard et al., 2012; Ait-Ouazzou et al., 2013). Although the mode of action of PEF also targets cell envelopes (García et al., 2005; Chueca et al., 2015), the survival benefits conferred by mutation against ICs and heat—but not against PEF—could have something to do with the repair and/or higher resistance displayed by cell envelope structures which are not targetted by PEF treatments. Along with damage to the bacterial membrane, heat also causes multitarget damages such as ribosome destabilization, enzyme denaturation, and DNA damage, among others (Mackey et al., 1991); carvacrol, moreover, could act as a transmembrane carrier of monovalent cations, fomenting the exchange of hydrogen and potassium ions between the cytoplasm and the external environment (Ultee et al., 2002) and could cause disruption to cellular metabolism and energy production (Chan et al., 2013). Consequently, the potential damage of cytoplasmic structures due to ICs and heat could lead to an absence of increase in PEF resistance (which seems to be exclusively and location-specifically related to membranes: cf. Chueca et al., 2015). In addition, one should note that the advantages conferred by mutations under certain conditions could represent a disadvantage under others—as observed for PEF resistance in CIT cells. Mutations could confer increased resistance to ICs and heat, leading to side-effects in PEF targets such as metabolic costs, higher exposure of the cytoplasmic membrane or a decreased ability to repair that type of damage.

The emergence of antibiotic resistance generally involves the development of specific mutations that enable bacteria to continue to grow in the presence of antibiotics (Cantón and Morosini, 2011); otherwise, it is connected with the selective advantage provided by naturally occurring mutants already present in the population before treatment (Collignon, 2002). Therefore, and, as previously researched in antibiotic-resistant strains (Renzoni et al., 2011), whole genome sequencing of derivative strains would permit the identification of single nucleotide polimorfisms (SNPs) and insertions or deletions (Indels) in those hyper-resistant strains in comparison with WT. Such valuable information would help to increase our knowledge of the mechanisms of bacterial inactivaction via carvacrol, citral, and (+)-limonene oxide. Furthermore, identification of these genotypic alterations would help us identify key structures and/or metabolic pathways involved in bacterial resistance to PEF. In addition, it is very likely that the results observed for the nonpathogenic *E. coli* strain used in this investigation would be similar to those that could occur in the case of pathogenic *E. coli* strains such as O157:H7.

Phase-contrast microscopial observation of hyper-resistant strains revealed filament formation in a small population (ca. 5% of total cells) of CAR and CIT strains (Figure 3). These changes in cell morphology appear to be a general response against stresses (Jones et al., 2013) such as treatments with antibiotics (Thi et al., 2011) and low temperatures (Visvalingam et al., 2012) which inhibit cell division or modify gene and protein expression in filamentous cells (Bereksi et al., 2002; Kieboom et al., 2006). Interestingly, previous publications have associated filamentation with slow growth such as that which we noted in the CAR and CIT strains (Figure 3). As observed for LIM cells, filamentation does not seem to be a pre-requisite for increased resistance. Nevertheless, the resistance displayed by these filamentous strains requires further research.

In addition, co-culture of CAR and CIT strains with the WT strain revealed a predominance of the original strain in absence of ICs (Figures 4A,C), indicating that these mutants would not outcompete the WT strain under optimal growth conditions. Consequently, although the emergence of stable strains that are hyper-resistant to food preservation technologies was confirmed in this study, it seems unlikely that mutant populations of CAR and CIT would take over the entire population under optimal growth conditions. Co-culture of LIM and WT strains nevertheless revealed a similar proportion of both strains in the final population (Figure 4F). Likewise, growth in presence of ICs favored the selection of CIT and LIM strains over the WT strain (Table 3), thus increasing the final proportion of hyper-resistant bacteria in the whole population. This could explain the high proportion of mutant cells we observed after 10 days of culturing cells in presence of sub-MIC concentrations of each IC (Table 1). Although ICs lowered the mutation frequency, incubation with sub-MIC concentrations of these antimicrobial compounds would act by selecting in favor of hyper-resistant strains generated during bacterial growth rather than by promoting their generation. As a result, food preservation treatments could be less effective if applied sequentially after treatment with sublethal concentrations of EOs or their ICs.

In conclusion, contrary to previous studies suggesting that EOs or their ICs had little impact on the development of antimicrobial resistance and susceptibility (McMahon et al., 2008; Hammer et al., 2012; Luz et al., 2012a,b; Gomes Neto et al., 2015), our results highlight the importance of preventing the emergence of hyper-resistant strains when using these compounds as antimicrobial agents. Thus, the application of low concentrations (sub-MIC) of EOs and their ICs aiming to slow bacterial multiplication prior to lethal preservation treatments should be avoided. This research demonstrates that the development of genotypic resistance to ICs has important consequences for the practical use of these antimicrobial compounds in food preservation.

## Author contributions

Conceived and designed the experiments: BC, RP, DG. Performed the experiments: BC, DB, NN. Analyzed the data: BC, DB, RP, DG. Wrote the paper: BC, RP, DG.

### Conflict of interest statement

The authors declare that the research was conducted in the absence of any commercial or financial relationships that could be construed as a potential conflict of interest.
